# Efficient Fuzzy C-Means Architecture for Image Segmentation

**DOI:** 10.3390/s110706697

**Published:** 2011-06-27

**Authors:** Hui-Ya Li, Wen-Jyi Hwang, Chia-Yen Chang

**Affiliations:** Department of Computer Science and Information Engineering, National Taiwan Normal University, Taipei 116, Taiwan; E-Mails: royalfay@gmail.com (H.-Y.L.); edifier5757@yahoo.com.tw (C.-Y.C.)

**Keywords:** fuzzy c-means, image segmentation, fuzzy clustering, fuzzy hardware, FPGA, reconfigurable computing, system on programmable chip

## Abstract

This paper presents a novel VLSI architecture for image segmentation. The architecture is based on the fuzzy c-means algorithm with spatial constraint for reducing the misclassification rate. In the architecture, the usual iterative operations for updating the membership matrix and cluster centroid are merged into one single updating process to evade the large storage requirement. In addition, an efficient pipelined circuit is used for the updating process for accelerating the computational speed. Experimental results show that the the proposed circuit is an effective alternative for real-time image segmentation with low area cost and low misclassification rate.

## 1. Introduction

Image segmentation plays an important role in computer vision and image analysis. The segmentation results can be used to identify regions of interest and objects in the scene, which is very beneficial to the subsequent image analysis or annotation. The fuzzy c-means algorithm (FCM) [1] is one of the most used technique for image segmentation. The accuracy of FCM is due to the employment of fuzziness for the clustering of each image pixel. This enables the fuzzy clustering methods to retain more information from the original image than the crisp or hard segmentation.

Although the original intensity-based FCM algorithm functions well on segmenting most noise-free images, it fails to segment images corrupted by noise, outliers and other imaging artifacts. The FCM with spatial constraint (FCM-S) algorithms [2–4] have been proposed to solve this problem by incorporating spatial information into original FCM objective function. However, as compared with the original FCM algorithm, the FCM-S algorithms have higher computational complexities for membership coefficients computation and centroid updating. In addition, similar to the original FCM algorithm, the size of membership matrix grows as the product of data set size and number of classes in the FCM-S. As a result, the corresponding memory requirement may prevent the algorithm from being applied to images with high dimension.

To accelerate the computational speed and/or reduce the memory requirement of the original FCM, a number of algorithms [5–8] have been proposed. These fast algorithms can be extended for the implementation of FCM-S. However, most of these algorithms are implemented by software, and only moderate acceleration can be achieved. In [9–11], hardware implementations of FCM are proposed. Nevertheless, the design in [9] is based on analog circuits. The clustering results therefore are difficult to be directly used for digital applications. Although the architecture shown in [10] adopts digital circuits, the architecture aims for applications with only two classes. In addition, it may be difficult to extend the architecture for the hardware implementation of FCM-S. The architecture presented in [11] operates with only a fixed degree of fuzziness *m* = 2 for the original FCM. The flexibility for selecting other degrees of fuzziness may be desired to further improve the FCM performance. In addition, similar to [10], the architecture presented in [11] cannot be directly used for the hardware implementation of FCM-S.

The objective of this paper is to present an effective digital FCM-S architecture for image segmentation. The architecture relaxes the restriction on the degree of fuzziness. The relaxation requires the employment of *n*-th root and division operations for membership coefficients and centroid computation. A pipeline implementation for the FCM-S therefore may be difficult. To solve the problem, in the proposed architecture, the *n*-th root operators and dividers are based on simple table lookup, multiplication and shift operations. Efficient pipeline circuits can then be adopted to enhance the throughput for fuzzy clustering.

To reduce large memory size for storing membership matrix, the proposed architecture combines the usual iterative updating processes of membership matrix and cluster centroid into a single updating process. In the architecture, the updating process is separated into three steps: pre-computation, membership coefficients updating, and centroid updating. The pre-computing step is used to compute and store information common to the updating of different membership coefficients. This step is beneficial for reducing the computational complexity for the updating of membership coefficients.

The membership updating step computes new membership coefficients based on a fixed set of centroids and the results of the pre-computation step. All the membership coefficients associated with a data point will be computed in parallel in this step. The computation time of the FCM-S therefore will be effectively expedited.

The centroid updating step computes the centroid of clusters using the current results obtained from the membership updating step. The weighted sum of data points and the sum of membership coefficients are updated incrementally here for the centroid computation. This incremental updating scheme eliminates the requirement for storing the entire membership coefficients.

The proposed architecture has been implemented on field programmable gate array (FPGA) devices [12] so that it can operate in conjunction with a softcore CPU [13]. Using the reconfigurable hardware, we are then able to construct a system on programmable chip (SOPC) system for image segmentation. The proposed architecture attain lower classification error rate in the presence of noise. In addition, compared with its software counterpart running on the 3.0 GHz Pentium D, our system has significantly lower computational time. All these facts demonstrates the effectiveness of the proposed architecture.

## 2. Preliminaries

We first give a brief review of the FCM algorithm. Let *X* = {*x*_1_, ..., *x_t_*} be a data set to be clustered by the FCM algorithm into *c* classes, where *t* is the number of data points in the design set. Each class *i*, 1 ≤ *i* ≤ *c*, is characterized by its centroid *v_i_*. The goal of FCM is to minimize the following cost function:

(1) $$J=\sum_{i=1}^{c}\sum_{k=1}^{t}u_{i,k}^{m}{\left\|x_{k}-v_{i}\right\|}^{2}$$

where *u_i,k_* is the membership of *x_k_* in class *i*, and *m* > 1 indicates the degree of fuzziness. The cost function *J* is minimized by a two-step iteration in the FCM. In the first step, the centroids *v*_1_, ..., *v_c_*, are fixed, and the optimal membership matrix {*u_i,k_*, *i* = 1, ..., *c*, *k* = 1, ..., *t*} is computed by

(2) $$u_{i,k}={\left(\sum_{j=1}^{c}{\left(||x_{k}-\upsilon_{i}||/||x_{k}-\upsilon_{j}||\right)}^{2/(m-1)}\right)}^{-1}$$

After the first step, the membership matrix is then fixed, and the new centroid of each class *i* is obtained by

(3) $$v_{i}=(\sum_{k=1}^{t}u_{i,k}^{m}x_{k})/(\sum_{k=1}^{t}u_{i,k}^{m})$$

A variant of FCM for image segmentation is FCM-S, whose objective function is [2]

(4) $$J=\sum_{i=1}^{c}\sum_{k=1}^{t}v_{i,k}^{m}{\left\|x_{k}-v_{k}\right\|}^{2}+\frac{\alpha }{Card(\Gamma )}\sum_{i=1}^{c}\sum_{k=1}^{t}u_{i,k}^{m}\sum_{j\in \Gamma }{\left\|x_{j}-v_{i}\right\|}^{2}$$

where Γ is the set of neighbors associated with *x_k_*, and the *Card*(Γ) is the cardinality of the set Γ. The parameter α determines the degree of penalty. The necessary conditions locally minimizing *J* are then given by

(5) $$u_{i,k}=\frac{{\left({\left\|x_{k}-v_{i}\right\|}^{2}+\frac{\alpha }{\mathrm{Card}(\Gamma )}\sum_{j\in \Gamma }{\left\|x_{j}-v_{i}\right\|}^{2}\right)}^{-1/(m-1)}}{\sum_{n=1}^{c}{\left({\left\|x_{k}-v_{n}\right\|}^{2}+\frac{\alpha }{\mathrm{Card}(\Gamma )}\sum_{j\in \Gamma }{\left\|x_{j}-v_{n}\right\|}^{2}\right)}^{-1/(m-1)}}$$

(6) $$v_{i}=\frac{\sum_{k=1}^{t}u_{i,k}^{m}(x_{k}+\frac{\alpha }{\mathrm{Card}(\Gamma )}\sum_{j\in \Gamma }x_{j})}{(1+\alpha )\sum_{k=1}^{t}u_{i,k}^{m}}$$

The disadvantages of Equations (5) and (6) are the high computational complexities for computing *u_i,j_* and *v_i_*. To accelerate the computation, observe from [3] that by simple manipulation, $\frac{1}{\mathrm{Card}(\Gamma )}\sum_{j\in \Gamma }{\left\|x_{j}-v_{i}\right\|}^{2}$ can be equivalently written as

(7) $$\frac{1}{\mathrm{Card}(\Gamma )}\sum_{j\in \Gamma }{\left\|x_{j}-v_{i}\right\|}^{2}=(\frac{1}{\mathrm{Card}(\Gamma )}{\sum_{j\in \Gamma }\left\|x_{j}-{\bar{x}}_{k}\right\|}^{2})\;+{\left\|{\bar{x}}_{k}-v_{i}\right\|}^{2}$$

where

(8) $${\bar{x}}_{k}=\frac{1}{\mathrm{Card}(\Gamma )}\sum_{j\in \Gamma }x_{j}$$

Note that *x̄_k_* can be computed in advance, and the minimization of *J* in Equation (4) is equivalent to the minimization of the following cost function.

(9) $$J=\sum_{i=1}^{c}\sum_{k=1}^{t}u_{i,k}^{m}{\left\|x_{k}-v_{i}\right\|}^{2}+\alpha \sum_{i=1}^{c}\sum_{k=1}^{t}u_{i,k}^{m}{\left\|{\bar{x}}_{k}-v_{i}\right\|}^{2}$$

Necessary conditions on *u_i,j_* and *v_i_* for locally minimizing *J* can be derived are follows.

(10) $$u_{i,k}=\frac{{\left({\left\|x_{k}-v_{i}\right\|}^{2}+\alpha {\left\|{\bar{x}}_{k}-v_{i}\right\|}^{2}\right)}^{-1/(m-1)}}{\sum_{j=1}^{c}{\left({\left\|x_{k}-v_{j}\right\|}^{2}+\alpha {\left\|{\bar{x}}_{k}-v_{j}\right\|}^{2}\right)}^{-1/(m-1)}}$$

(11) $$v_{i}=\frac{\sum_{k=1}^{t}u_{i,k}^{m}(x_{k}+\alpha {\bar{x}}_{k})}{(1+\alpha )\sum_{k=1}^{t}u_{i,k}^{m}}$$

The FCM and FCM-S algorithms requires large number of floating point operations. Moreover, from Equations (1), (3), (10) and (11), it follows that the membership matrix needs to be stored for the computation of cost function and centroids. As the size of the membership matrix grows with the product of *t* and *c*, the storage size required for the FCM may be impractically large when the data set size and/or the number of classes become high.

## 3. The Proposed Architecture

The goal of the proposed architecture is to implement the FCM-S algorithm in hardware. The architecture is based on a novel pipeline circuit to provide high throughput for fuzzy clustering. It is also able to eliminate the requirement for storing the large membership matrix for the computation of cost function and centroids.

**Figure 1.**
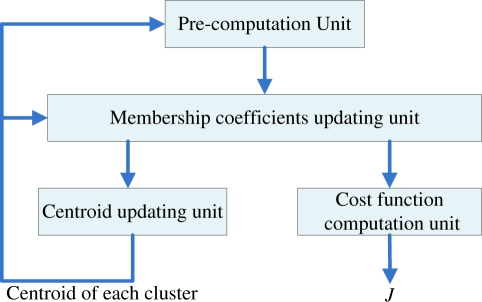
The basic VLSI architecture for realizing the proposed FCM algorithm.

As shown in Figure 1, the proposed FCM-S architecture can be decomposed into four units: the pre-computation unit, the membership coefficients updating unit, centroid updating unit and cost function computation unit. These four units will operate concurrently in pipeline fashion for the clustering process.

For sake of simplicity, the architecture of these four units for the original FCM are presented first. Their extensions to the FCM-S will then be discussed.

### 3.1. Pre-Computation Unit for Original FCM

The pre-computation unit is used for reducing the computational complexity of the membership coefficients calculation. Observe that *u_i,k_* in Equation (2) can be rewritten as

(12) $$u_{i,k}=\;{\left\|x_{k}-v_{i}\right\|}^{-2/(m-1)}P_{k}^{-1}$$

where

(13) $$P_{k}=\sum_{j=1}^{c}{\left(1/{\left\|x_{k}-v_{j}\right\|}^{2}\right)}^{1/(m-1)}$$

Given *x_k_* and centroids *v*_1_, ..., *v_c_*, membership coefficients *u*_1,_*_k_*, ..., *u_c,k_* have the same *P_k_*. Therefore, the complexity for computing membership coefficients can be reduced by calculating *P_k_* in the pre-computation unit. Without loss of generality, the degree of fuzziness *m* can be expressed as

(14) m=a/b

where both *a* and *b* are integers. Because *m* should be larger than 1, it follows that *a* > *b* > 0. Let

(15) r=b,n=a−b

We then can rewrite Equation (13) as

(16) $$P_{k}=\sum_{j=1}^{c}{\left(\left\|x_{k}-v_{j}\right\|\right)}^{-2r/n}$$

Based on Equation (16), we see that the *n*-th root operation is required for the implementation of *p_k_*. In the proposed architecture, a novel *n*-th root circuit is adopted so that *P_k_* can be implemented in a pipelined fashion. In the proposed *n*-th root circuit, the goal is to compute $\sqrt[{n}]{Y}$, where

$$Y=1+2^{-1}y_{1}+2^{-2}y_{2}+\ldots +2^{-(2q-1)}y_{2q-1}.$$

That is, *Y* is a 2*q*-bits real number such that 1 < *Y* < 2. We separate *Y* into two portions *Y_h_* and *Y_l_* as shown below

(17) $$Y_{h}=1+2^{-1}y_{1}+2^{-2}y_{2}+\ldots +2^{-(q-1)}y_{q-1}$$

(18) $$Y_{l}=2^{-(q+1)}y_{q+1}+2^{-(q+2)}y_{q+2}+\ldots +2^{-(2q-1)}y_{2q-1}$$

For the sake of simplicity, we first consider the computation of $\sqrt{Y}$. Observe that

$$\sqrt{Y}=\frac{Y}{{\left(Y_{h}+Y_{l}\right)}^{1/2}}=\frac{Y/Y_{h}^{1/2}}{{\left(1+Y_{l}/Y_{h}\right)}^{1/2}}=\frac{Y}{Y_{h}^{1/2}}(1-\frac{Y_{l}}{2Y_{h}}+\frac{3Y_{l}^{2}}{8Y_{h}^{2}}\ldots )$$

By retaining the first two terms of the Taylor series, $\sqrt{Y}$ can be approximated by

$$\sqrt{Y}\approx \frac{Y}{Y_{h}^{1/2}}(1-\frac{Y_{l}}{2Y_{h}})=\frac{Y(Y_{h}-Y_{l}/2)}{Y_{h}^{3/2}}$$

From Equations (17) and (18), we conclude that *Y_h_* > 2*^q^Y_l_*. Therefore, the maximum error of the approximation is less than 2^−2^*^q^*. Following the same procedure, it can also be found that

$$\sqrt[{3}]{Y}=\frac{Y}{{\left(Y_{h}+Y_{l}\right)}^{2/3}}=\frac{Y/Y_{h}^{2/3}}{{\left(1+Y_{l}/Y_{h}\right)}^{2/3}}=\frac{Y}{Y_{h}^{2/3}}(1-\frac{2Y_{l}}{3Y_{h}}+\ldots )\approx \frac{Y(Y_{h}-2Y_{l}/3)}{Y_{h}^{5/3}}$$

These results can be extended for any *n* ≥ 2 as follows:

(19) $$\sqrt[{n}]{Y}\approx \frac{Y(Y_{h}-(n-1)Y_{l}/n)}{Y_{h}^{(2n-1)/n}}$$

**Figure 2.**
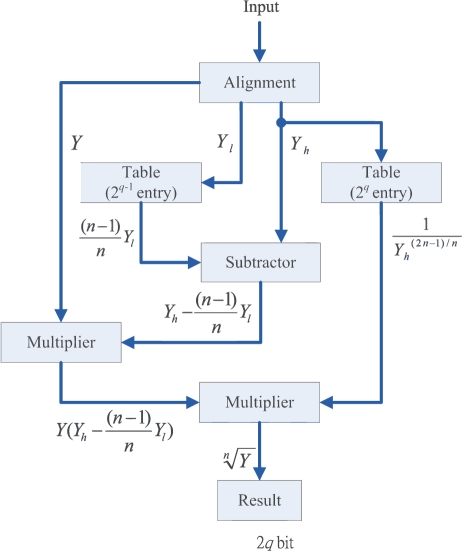
The architecture of *n*-th root unit.

The *n*-th root circuit based on Equation (19) is shown in Figure 2, which consists of two tables, two multipliers, and one adder. The tables store (*n* − 1)*Y_l_*/*n* and $Y_{h}^{(2n-1)/n}$ for all the possible values of *Y_l_* and *Y_h_*. Although it is possible to construct a table directly for $\sqrt[{n}]{Y}$, the number of entries in the table would be 2^2^*^q^*^−1^ because *Y* contains 2*q* bits. By contrast, both *Y_h_* and *Y_l_* consist of only *q* bits. The number of entries in each table shown in Figure 2 is only 2*^q^*^−1^. Consequently, the proposed circuit is able to perform fast and accurate computation while maintaining low area cost.

**Figure 3.**
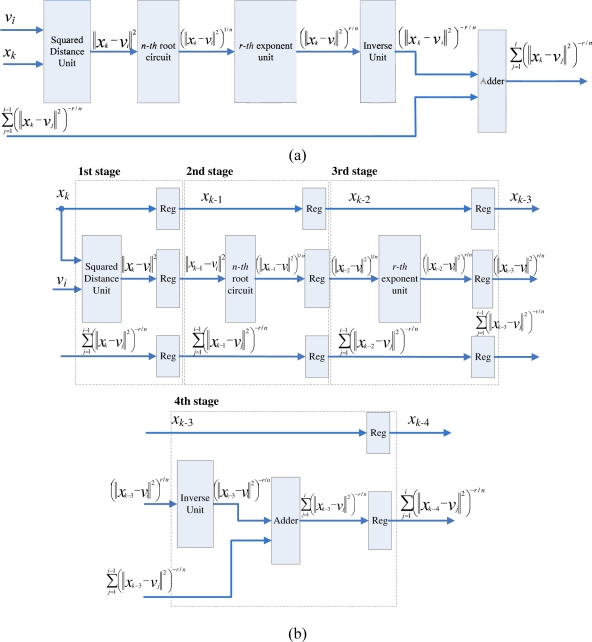
The circuit for evaluating (||*x_k_* − *v_j_*||)^−2*r/n*^. **(a)** Basic circuit; **(b)** 4-stage pipeline architecture.

Observe from Equation (16) that the computation of *P_k_* can be separated into *c* terms, where the *j*-th term involves the computation of (||*x_k_* − *v_j_*||)^−2*r/n*^. The basic circuit for calculating (||*x_k_* − *v_j_*||)^−2*r/n*^ is shown in Figure 3(a). In addition to the *n*-th root circuit, it contains squared distance unit, *r*-th power unit and inverse operation unit. Both the squared distance unit and the *r*-th power circuit are based on multipliers. Similar to the *n*-th root circuit, the inverse operation circuit is also based on tables, multipliers and adders [14].

The basic circuit for calculating (||*x_k_* − *v_j_*||)^−2*r/n*^ can be separated into a number of stages for pipeline implementation. Figure 3(b) shows an example for 4-stage pipeline implementation. It can be observed from the figure that two training vectors *x_k_*, *x_k_*_−1_, *x_k_*_−2_ and *x_k_*_−3_ are operated concurrently in the pipeline, where the first, second, third and fourth stages are used for computing ||*x_k_* − *v_j_*||^2^ and (||*x_k_*_−1_ − *v_j_*||^2^)^1/*n*^, (||*x_k_*_−2_ − *v_j_*||^2^)*^r/n^*, and (||*x_k_*_−3_ − *v_j_*||^2^)^−^*^r/n^*, respectively.

**Figure 4.**
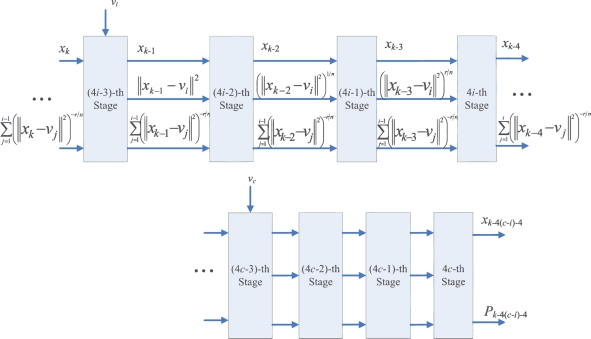
Architecture of Pre-computation unit.

To compute *P_k_*, the accumulation of the results of (||*x_k_* − *v_j_*||)^−2^*^r/n^* for *j* = 1, ..., *c*, is required. This can be accomplished by the employment of an accumulator at the fourth stage, as shown in Figure 3(b). Consequently, we can cascade the circuit shown in Figure 3(b) for calculating each (||*x_k_* − *v_j_*||)^−2^*^r/n^*, *j* = 1, ..., *c*, to a 4*c*-stage pipeline for computing *P_k_*. Figure 4 shows the architecture of the pipeline. The (4*i* − 1)-th stage, (4*i* − 2)-th stage, (4*i* − 3)-th stage, and (4*i* − 4)-th stage of the pipeline are the first, second, third and fourth stage of the circuit in Figure 3(b), respectively.

When *x_k_* enters the (4*i* − 1)-th stage, the accumulator at the 4*i*-th stage receives the sum of (||*x_k_*_−4_ − *v*_1_||^2^)^−^*^r/n^*, ..., (||*x_k_*_−4_ − *v_i_*_−1_||^2^)^−^*^r/n^*, from its precedent accumulator. It then adds the results of (||*x_k_*_−4_ − *v_i_*||^2^)^−^*^r/n^* to the sum, and then propagates the results to the subsequent stages. As the computation at the 4*c*-th stage for data point *x_k_* is completed, the output of the pre-computation unit is *P_k_*.

### 3.2. Membership Coefficients Updating Unit for Original FCM

The membership coefficients updating unit receives the *P_k_* value from the pre-computation unit, and then compute $u_{i,k}^{m}$ for *i* = 1, ..., *c*, concurrently. From Equations (12), (14) and (15), it follows that

(20) $$u_{i,k}^{m}={\left({\left({\left\|x_{k}-v_{i}\right\|}^{2}\right)}^{1/n}P_{k}^{1/r}\right)}^{-(n+r)}$$

**Figure 5.**
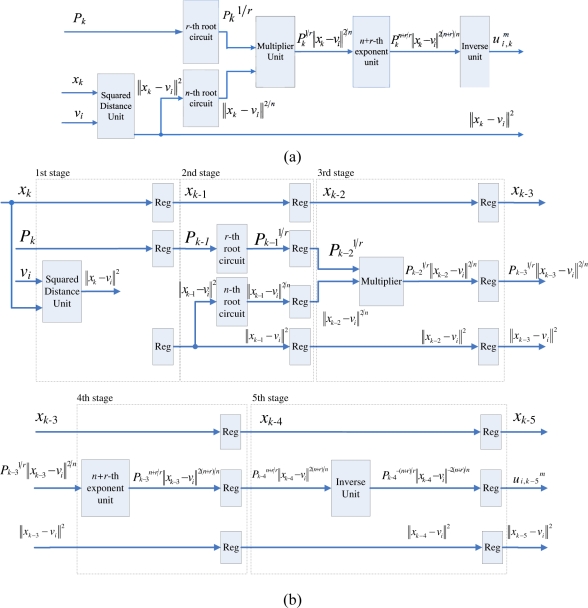
The circuit for evaluating $u_{i,k}^{m}$. **(a)** The basic circuit; **(b)** 5-stage pipeline architecture.

The basic circuit for computing $u_{i,k}^{m}$ is shown in Figure 5(a). Based on Equation (20), it follows that the circuit contains squared distance unit, *r*-th root and *n*-th root circuits, (*n* + *r*)-th power circuit, and inverse unit. From the figure, we observe that ||*x_k_* − *v_i_*||^2^ is first computed. This is accomplished by the squared distance unit. Following that, the *r*-th root circuit and *n*-th root circuit are used for computing $P_{k}^{1/r}$ and (||*x_k_* − *v_i_*||^2^)^1/^*^n^*, respectively. The (*n* + *r*)-th power circuit is then adopted for computing ${\left({\left({\left\|x_{k}-v_{i}\right\|}^{2}\right)}^{1/n}P_{k}^{1/r}\right)}^{\left(n+r\right)}$. Finally, the inverse unit is employed for evaluating $u_{i,k}^{m}$. Similar to the pre-computation unit, the basic circuit for computing $u_{i,k}^{m}$ can also be implemented in a pipeline fashion. An example of 5-stage pipeline implementation is shown in Figure 5(b).

Because $u_{i,k}^{m}$ for *i* = 1, ..., *c*, can be computed in parallel, there are *c* identical modules in the membership coefficients updating unit. The module *i* in the unit is used for computing $u_{i,k}^{m}$. The architecture of the module *i* of the unit is shown in Figure 5(b). Therefore, in the membership coefficients updating unit, the $u_{i,k}^{m}$ for *i* = 1, ..., *c*, can be obtained in 5 clock cycles after *x_k_* is presented at the input of the unit.

### 3.3. Centroid Updating Unit for Original FCM

The centroid updating unit incrementally computes the centroid of each cluster. The major advantage for the incremental computation is that it is not necessary to store the entire membership coefficients matrix for the centroid computation. To elaborate this fact, we first define the incremental centroid for the *i*-th cluster up to data point *x_k_* as

(21) $$v_{i}(k)=(\sum_{n=1}^{k}u_{i,n}^{m}x_{n})/(\sum_{n=1}^{k}u_{i,n}^{m})$$

**Figure 6.**
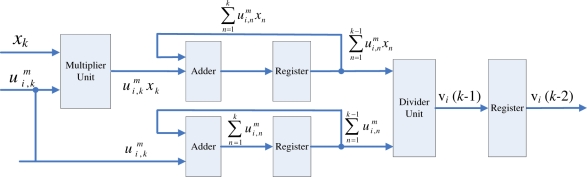
The basic circuit for calculating *v_i_*(*k*).

When *k* = *t*, *v_i_*(*k*) then is identical to the actual centroid *v_i_* given in Equation (3). Based on Equation (21), it can be observed that the computation of *v_i_*(*k*) is based on $\sum_{n=1}^{k-1}u_{i,n}^{m}x_{n}$, $\sum_{n=1}^{k-1}u_{i,n}^{m},u_{i,k}^{m}$ and *x_k_*. To compute *v_i_*(*k*), as shown in Figure 6, two accumulators can be used for storing $\sum_{n=1}^{k-1}u_{i,n}^{m}x_{n}$, and $\sum_{n=1}^{k-1}u_{i,n}^{m}$, respectively. When $u_{i,k}^{m}$ and *x_k_* are received, both $\sum_{n=1}^{k}u_{i,n}^{m}x_{n}$ and $\sum_{n=1}^{k}u_{i,n}^{m}$ can be obtained by adding $u_{i,k}^{m}x_{k}$ and $u_{i,k}^{m}$ to the two accumulators, respectively. Based on the output of these two accumulators, *v_i_*(*k*) can then be computed by the divider. In the incremental computation scheme, it is therefore not necessary to store membership coefficients $u_{i,n}^{m}$ and training vectors *x_n_*, *n* = 1, ..., *k* − 1, for the computation of *v_i_*(*k*). The two accumulators already have the partial results $\sum_{n=1}^{k-1}u_{i,n}^{m}x_{n}$, and $\sum_{n=1}^{k-1}u_{i,n}^{m}$ for the computation. In addition, after adding $u_{i,k}^{m}x_{k}$ and $u_{i,k}^{m}$ to the two accumulators, both $u_{i,k}^{m}$ and *x_k_* are no longer required in the circuit. Based on the updated outputs of the accumulators $\sum_{n=1}^{k}u_{i,n}^{m}x_{n}$, and $\sum_{n=1}^{k}u_{i,n}^{m}$, and new incoming membership coefficients and training vectors, we are able to compute *v_i_*(*l*) for *l* > *k*. Thus, no membership coefficients matrix is needed in our design.

The centroid updating unit contains *c* identical modules. All modules operate concurrently. The goal of each module *i* is to compute *v_i_*(*k*). Therefore, each module *i* is implemented by the circuit shown in Figure 6. Note that the *v_i_*(*k*) at the output is only the incremental centroid. Therefore, *v_i_* used by the pre-computation unit and membership coefficients updating unit will not be replaced by *v_i_*(*k*) until the *v_i_*(*t*) is obtained.

### 3.4. Cost Function Computation Unit for Original FCM

As shown in Figure 1, the cost function computation unit operates in parallel with the centroid updating unit. Similar to the centroid updating unit, the cost function unit incrementally computes the cost function *J*. Define the incremental cost function *J*(*k*) up to data point *x_k_* as

(22) $$J(k)=\sum_{i=1}^{c}\sum_{n=1}^{k}u_{i,n}^{m}{\left\|x_{n}-u_{i}\right\|}^{2}$$

**Figure 7.**
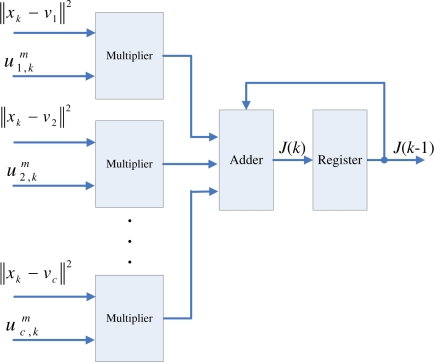
The architecture of cost function computation unit.

As shown in Figure 7, the circuit receives $u_{i,k}^{m}$ and ||*x_k_* − *v_i_*||^2^ *i* = 1, ..., *c*, from the membership coefficients updating unit. The products $u_{i,k}^{m}{\left\|x_{k}-v_{i}\right\|}^{2}$, *i* = 1, ..., *c* are then accumulated for computing *J*(*k*) in Equation (22).

When *k* = *t*, *J_k_* then is identical to the actual cost function *J* given in Equation (1). Therefore, the output of the circuit becomes *J* as the cost function computations for all the training vectors are completed.

### 3.5. FCM-S Architecture

**Figure 8.**
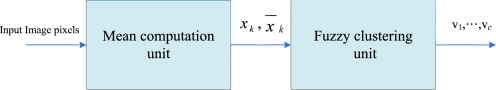
The FCM-S architecture.

Figure 8 shows the architecture of FCM-S, which consists of two units: the mean computation unit and the fuzzy clustering unit. The goal of the mean computation unit is to evaluate the mean value *x̄_k_* defined in Equation (8). The main architecture of FCM-S is the fuzzy clustering unit, which computes the membership coefficients and centroids of FCM-S. Therefore, our discussion in this subsection will focus on the fuzzy clustering unit of the FCM-S. Using Equations (14) and (15), we can rewrite the membership coefficients of FCM-S defined in Equation (10) as

(23) $$u_{i,k}^{m}={\left({\left({\left\|x_{k}-v_{i}\right\|}^{2}+\alpha {\left\|{\bar{x}}_{k}-v_{i}\right\|}^{2}\right)}^{1/n}P_{k}^{1/r}\right)}^{-(n+r)}$$

where

(24) $$P_{k}=\sum_{j=1}^{c}{\left({\left\|x_{k}-v_{j}\right\|}^{2}+\alpha {\left\|{\bar{x}}_{k}-v_{j}\right\|}^{2}\right)}^{-r/n}$$

**Figure 9.**
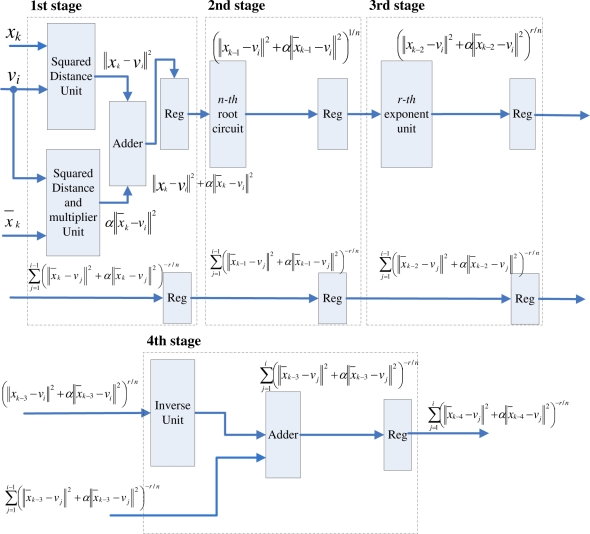
The circuit for evaluating (||*x_k_* − *v_j_*||^2^ + α||*x̄_k_* − *v_j_*||^2^)^−^*^r/n^*.

Similar to the original FCM, it follows from Equation (24) that the computation of *P_k_* can also be separated into *c* terms, where the *j*-th term involves the computation of (||*x_k_* − *v_j_*||^2^ + α||*x̄_k_* − *v_j_*||^2^)^−^*^r/n^*. Figure 9 shows the architecture for the computation of each (||*x_k_* − *v_j_*||^2^ + α||*x̄_k_* − *v_j_*||^2^)^−^*^r/n^*. From Figure 9, we see that the architecture can also be implemented as a 4-stage pipeline, similar to that shown in Figure 3(b) for computing (||*x_k_* − *v_j_*||)^−2^*^r/n^*. Therefore, the pre-computation unit for FCM-S can be realized as a 4*c* stage pipeline shown in Figure 4.

Both pipelines in Figures 3(b) and 9 have similar architectures. The only difference is that the first stage of the pipeline in Figure 9 has higher area and computational complexities. There are two squared distance calculation units and one adder at the first stage of the pipeline in Figure 9. By contrast, there is only one squared distance unit at the first stage of the pipeline in Figure 3(b). In fact, Observe from Equations (16) and (24) that the *P_k_* for FCM-S can be viewed as the generalized version of *P_k_* for original FCM by replacing the squared distance ||*x_k_* − *v_j_*||^2^ in Equation (16) with ||*x_k_* − *v_j_*||^2^ + α||*x̄_k_* − *v_j_*||^2^. Hence, the pipeline in Figure 9 is also an extension of that in Figure 3(b) by replacing the simple squared distance calculation ||*x_k_* − *v_j_*||^2^ at the first stage with ||*x_k_* − *v_j_*||^2^ + α||*x̄_k_* − *v_j_*||^2^.

**Figure 10.**
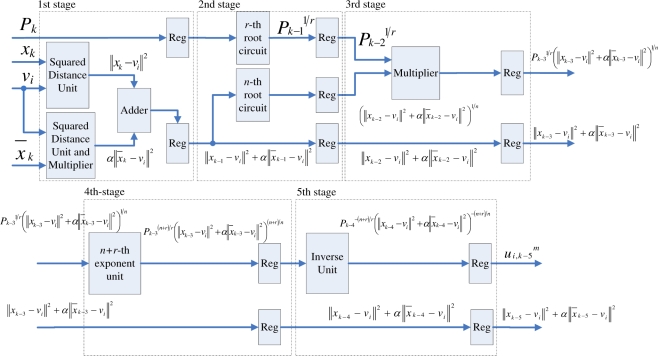
The circuit for evaluating $u_{i,k}^{m}$ for FCM-S.

**Figure 11.**
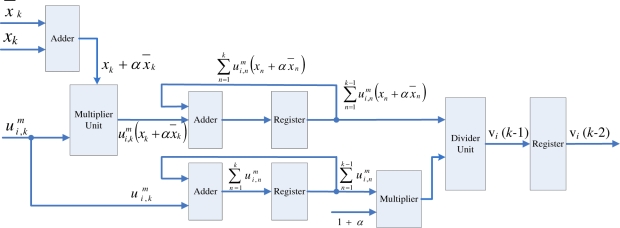
The circuit for calculating *v_i_*(*k*) for FCM-S.

**Figure 12.**
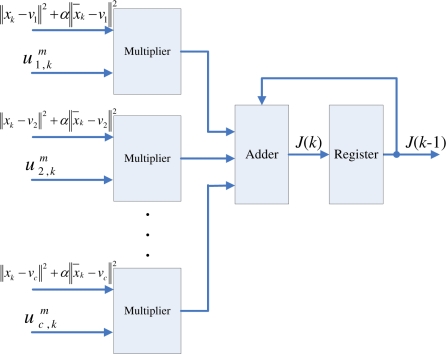
The circuit for calculating cost function *J*(*k*) for FCM-S.

Figures 10–12 depict the architecture for membership coefficients updating, centroids updating and cost function computation for FCM-S based on Equations (9), (11) and (23), respectively. Similar to the original FCM algorithm, the proposed FCM-S architecture computes the centroids and cost function incrementally. In the FCM-S, the incremental centroid for the *i*-th cluster up to data point *x_k_* is defined as

(25) $$v_{i}(k)=(\sum_{n=1}^{k}u_{i,n}^{m}(x_{n}+\alpha {\bar{x}}_{n}))/((1+\alpha )(\sum_{n=1}^{k}u_{i,n}^{m})).$$

In addition, the incremental cost function *J*(*k*) up to data point *x_k_* is defined as

(26) $$J(k)=\sum_{i=1}^{c}\sum_{n=1}^{k}u_{i,n}^{m}({\left\|x_{n}-v_{i}\right\|}^{2}+\alpha {\left\|{\bar{x}}_{k}-v_{j}\right\|}^{2}).$$

As shown in Figures 11 and 12, the goals of the centroids updating unit and the cost function computation unit are to compute *v_i_*(*k*) and *J*(*k*), respectively. As *k* = *t*, the *v_k_*(*i*) and *J*(*k*) in Equations (25) and (6) will becomes *v*(*i*) in Equation (11) and *J* in Equation (9), respectively.

We can view the membership coefficients, centroids and cost function for FCM-S as the extension of those for original FCM by replacing ||*x_k_* − *v_j_*||^2^ with ||*x_k_* − *v_j_*||^2^ + α||*x̄_k_* − *v_j_*||^2^. Therefore, the membership coefficients updating unit, centroids updating unit and cost function computation unit for FCM-S also have similar architectures to those of their counterparts in original FCM. The circuits in FCM-S require only additional squared distance unit and adder for computing ||*x_k_* − *v_j_*||^2^ + α||*x̄_k_* − *v_j_*||^2^.

### 3.6. The SOPC System Based on the Proposed Architecture

**Figure 13.**
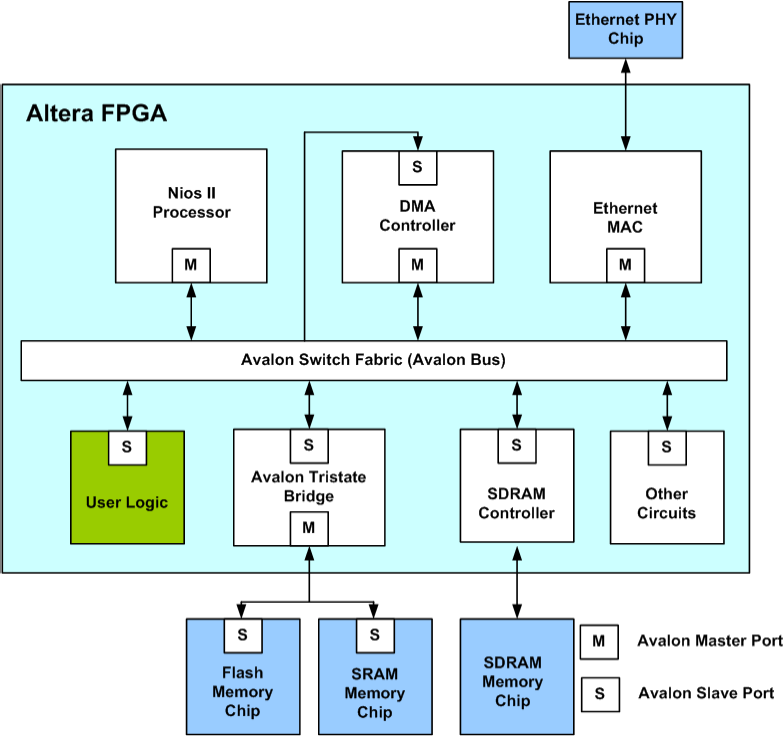
The SOPC system for FCM-based image segmentation.

The proposed architecture is used as a custom user logic in a SOPC system consisting of softcore NIOS CPU, DMA controller and SDRAM, as depicted in Figure 13. The set of training vectors is stored in the SDRAM. The training vectors are then delivered to the proposed circuit by the DMA controller. The softcore NIOS CPU is running a simple software for FCM. It does not participate in the partitioning and centroid computation processes. The software only activates the DMA controller for the delivery of training vectors. The CPU then receives the overall distortion of clustering from the proposed circuit after the completion of DMA operation. The same DMA operation for delivering the training data to the proposed circuit will be repeated until the cost function *J* converges. The CPU then collects the centroid of each cluster from the proposed circuit as the clustering results.

## 4. Experimental Results

This section presents some numerical results of the proposed FCM-S architecture for image segmentation. The design platform of our system is Altera Quartus II with SOPC Builder and NIOS II IDE. The target FPGA device for the hardware implementation is Altera Stratix II EP2S60 [15]. All the images considered in the experiments in this section are of size 320 × 320. Each pixel of the images is corrupted by i.i.d. noise with uniform distribution in the interval [−*b*, *b*].

For sake of brevity, the images considered in this section are gray-level images. Each data point *x_k_* represents a pixel with gray level values in the range between 0 and 255. For color images, each pixel *x_k_* becomes a vector consisting of three color components: red, green and blue. In the proposed architecture, each data point *x_k_* can be a scalar or a vector. Therefore, the proposed architecture can be directly applied to color image segmentation by implementing *x_k_* as a 3-dimension vector.

The performance of the image segmentation is measured by segmentation error rate, which is equal to the number of misclassified pixels divided by the total number of pixels. Table 1 shows the segmentation error rate of the original FCM algorithm and the FCM-S algorithm for various *b* values for the images “Apple” and “Strawberry”. The number of classes is *c* = 2. The degree of membership is given by *m* = 1.5.

**Figure 14.**
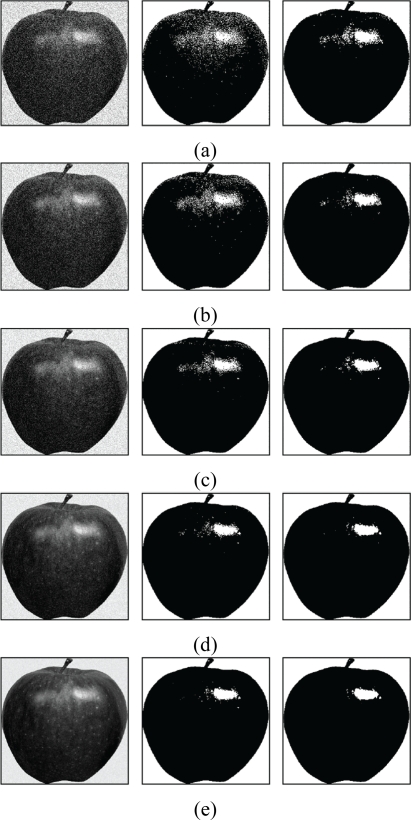
Segmentation results of the image “Apple”. (**a**) *b* = 80; (**b**) *b* = 60; (**c**) *b* = 40; (**d**) *b* = 20; (**e**) *b* = 10. The first column represents corrupted images, the second column shows results using FCM algorithm and the third column reveals the segmentation performance of FCM-S algorithm.

**Figure 15.**
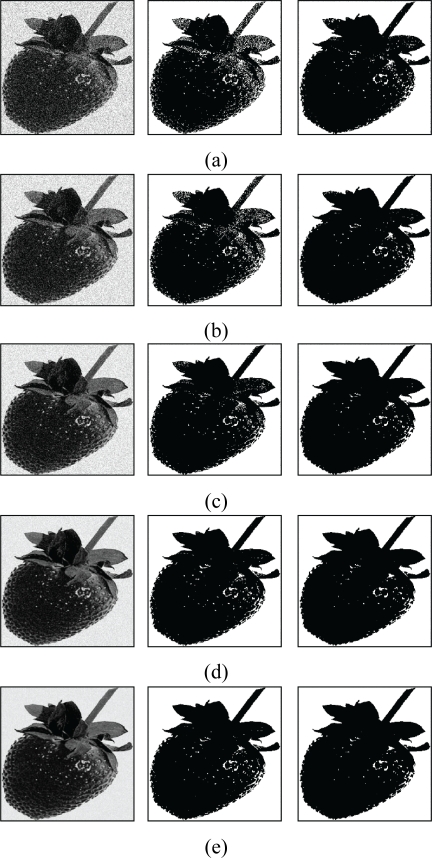
Segmentation results of the image “Strawberry”. **(a)** *b* = 80; **(b)** *b* = 60; (**c**) *b* = 40; **(d)** *b* = 20; **(e)** *b* = 10. The first column represents corrupted images, the second column shows results using FCM algorithm and the third column reveals the segmentation performance of FCM-S algorithm.

**Figure 16.**
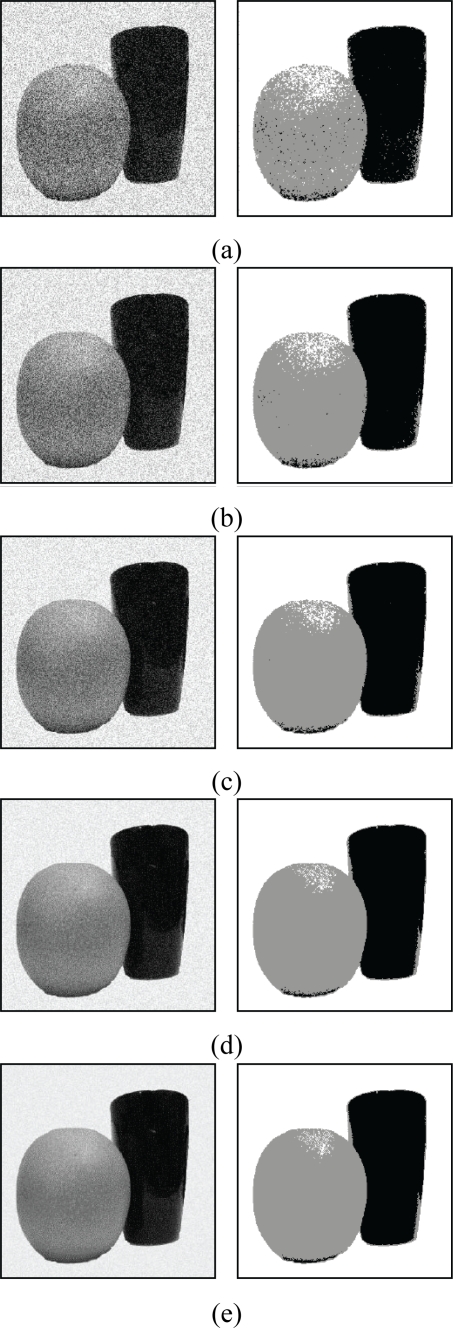
Segmentation results of the image “Pear & Cup”. **(a)** *b* = 80; **(b)** *b* = 60; (**c**) *b* = 40; **(d)** *b* = 20; **(e)** *b* = 10. The first column represents corrupted images, and the second column shows results using the FCM-S algorithm.

From the Table 1, we see that FCM-S has lower segmentation error rate as compared with the original FCM. In addition, their gap in the error rate increases as the noise becomes larger. The FCM-S is able to attain lower segmentation error rate because the spatial information is used during the training process. However, in the original FCM, the spatial information is not used. Figures 14 and 15 show the segmentation results of the images “Apple” and “Strawberry” for various *b* values. Table 2 and Figure 16 show the segmentation error rate and segmentation results of FCM-S for the image “Pear & Cup”, respectively. The image contains three classes (*i.e.*, *c* = 3). We can see from Table 2 and Figure 16 that the FCM-S performs well for the noisy images with more than two classes.

Table 3 compares the segmentation error rate of the FCM-S for the images “Apple” and “Strawberry” for various degree of fuzziness *m*. It can be observed from the table that the FCM-S with *m* = 1.5 has lowest segmentation error rate. In fact, the segmentation error rate of FCM-S with *m* = 1.5 is lower than that of FCM-S with *m* = 2.0 for all the *b* values considered in this experiment. Note that when *m* = 2.0, the FCM circuit design can be simplified. In this case, *n* = *r* = 1. Therefore, no *n*-th root and *r*-th power circuits are required. Table 4 shows the area cost of FCM-S for various *m* values with *c* = 2. It is not surprising to see that FCM-S with *m* = 2.0 consumes the least hardware resources. In fact, when *m* = 2, the number of adaptive look-up tables (ALUTs) used by the architecture is only 9% of that of the target FPGA device. Consequently, when hardware resources are the important concern, we can select the degree of fuzziness as *m* = 2. On the other hand, when more accurate segmentation is desired, we can adopt the proposed architecture with other *m* values at the expense of possible increase in hardware resources consumption.

Table 5 compares the hardware resource consumption of the original FCM with FCM-S with *c* = 2. Given the same *m* value, we can see from Table 5 that the FCM-S only has slightly higher area costs as compared with FCM. The FCM-S architecture has higher hardware costs because it needs more squared distance computation circuits, multipliers and/or adders at the pre-computation unit, membership coefficients updating unit and centroid computation unit.

The proposed architecture is adopted as an hardware accelerator of a NIOS II softcore processor. Table 6 shows the area costs of the entire SOPC system based on the proposed FCM-S architectures with different *m* values. Because the NIOS II processor also consumes hardware resources, the consumptions of ALUT, embedded memory bits and DSP blocks of the entire SOPC are higher than those of FCM-S architecture, as shown in Tables 4 and 6. Nevertheless, the number of ALUTs, embedded memory bits and DSP blocks used by the SOPC system are lower than 40% of those of the target FPGA device.

The computation speed of the FCM and FCM-S architectures and their software counterparts are shown in Table 7 for various *m* values. The softcore processor of the SOPC systems are operating at 50 MHz. The software implementation of FCM and FCM-S algorithms are based on 3.0 GHz Pentium D processor with 2.0 Gbyte DDR2. Because the FCM-S algorithm has higher computation complexities, the algorithm has longer computation time as compared with the original FCM. The increase in computation time may be large for software implementation. For example, when *m* = 1.5, the computation time of FCM and FCM-S algorithms implemented by software are 152.9 ms and 196.09 ms, respectively. The employment of FCM-S in software therefore results in 28.28% increase in computation time. By contrast, the computation time of FCM and FCM-S algorithms implemented by hardware are 0.5703 ms and 0.5815 ms, respectively. Hence, only 1.96% increase in computation time is observed when FCM architecture is replaced by FCM-S architecture. It can also be observed from Table 6 that the FCM and FCM-S architectures have high speedup over its software counterparts. The proposed architectures have high speedup because the architectures are based on high throughput pipelines. In particular, when *m* = 2.0, the speedup is 342.51. The proposed architecture therefore is well-suited for realtime segmentation of noisy images with low error rate and low hardware resource consumption.

## 5. Concluding Remarks

The proposed FCM-S architecture has been found to be effective for image segmentation. To lower the segmentation error rate, in the proposed architecture, the spatial information is used during the FCM training process. The architecture can also be designed for different values of degree of fuzziness to further improve the segmentation results. In addition, the architecture employs high throughput pipeline to enhance the computation speed. The *n*-th root circuits and inverse operation circuits in the architecture are designed by simple lookup tables and multipliers for lowering the hardware resource consumption. Experimental results reveal that the proposed architecture is able to achieve segmentation error rate down to 1.9% for noisy images. In addition, the SOPC architecture attains speedup up to 342.51 over its software counterpart. The proposed architecture therefore is an effective alternative for applications requiring realtime image segmentation and analysis.

## Figures and Tables

**Table 1.** The segmentation error rate of the original FCM algorithm and the FCM-S algorithm for various *b* values for the images “Apple” and “Strawberry”.

***b* values**	**10**	**20**	**40**	**60**	**80**
FCM for image “Apple”	0.020	0.022	0.028	0.041	0.074
FCM-S for image “Apple”	0.019	0.020	0.021	0.024	0.029
FCM for image “Strawberry”	0.024	0.025	0.033	0.050	0.066
FCM-S for image “Strawberry”	0.020	0.021	0.022	0.025	0.029

**Table 2.** The segmentation error rate of the FCM-S algorithm for various *b* values for the images “Pear & Cup”.

***b* values**	**10**	**20**	**40**	**60**	**80**
FCM-S for image “Pear & Cup”	0.023	0.024	0.033	0.039	0.054

**Table 3.** The segmentation error rate of the FCM-S algorithm for various *m* values for the images “Apple” and “Strawberry”.

***b* values**	**10**	**20**	**40**	**60**	**80**
*m* = 1.5 for image “Apple”	0.019	0.020	0.021	0.024	0.029
*m* = 2.0 for image “Apple”	0.020	0.020	0.022	0.025	0.031
*m* = 2.5 for image “Apple”	0.020	0.021	0.023	0.027	0.031
*m* = 1.5 for image “Strawberry”	0.020	0.021	0.022	0.025	0.029
*m* = 2.0 for image “Strawberry”	0.021	0.022	0.023	0.026	0.030
*m* = 2.5 for image “Strawberry”	0.022	0.022	0.023	0.027	0.031

**Table 4.** Hardware resource consumption of the FCM-S architecture with different *m* values.

***m* values**	**ALUTs**	**Embedded memory bits**	**DSP blocks**
1.5	8246 (17%)	63684 (3%)	72 (25%)
1.75	9256 (19%)	112048 (4%)	100 (35%)
2	4152 (9%)	38944 (2%)	20 (7%)
2.25	8500 (18%)	112048 (4%)	100 (35%)
2.5	9106 (19%)	112048 (4%)	80 (28%)

**Table 5.** Comparisons of hardware resource consumption of the original FCM and FCM-S architectures for different *m* values.

***m* values**	**ALUT**		**Embedded memory bits**		**DSP blocks**	
	**FCM**	**FCM-S**	**FCM**	**FCM-S**	FCM	FCM-S
1.5	5270	8246	63684	63684	56	72
2.0	3468	4152	38944	38944	20	20
2.5	8371	9106	112048	112048	80	80

**Table 6.** Hardware resource consumption of the entire SPOC system based on the FCM-S architecture with different *m* values.

***m* values**	**ALUTs**	**Embedded memory bits**	**DSP blocks**
1.5	17960 (37%)	955936 (38%)	80 (28%)
1.75	19415 (40%)	1004336 (39%)	108 (38%)
2	14214 (29%)	931488 (37%)	28 (10%)
2.25	19355 (40%)	1004336 (39%)	108 (38%)
2.5	19234 (40%)	1004336 (39%)	88 (31%)

**Table 7.** Comparisons of computation speed of the original FCM and FCM-S architectures for different *m* values.

***m* values**	**FCM**			**FCM-S**		
	**Software**	**Hardware**	**Speedup**	**Software**	**Hardware**	**Speedup**
1.5	152.9 ms	0.5703 ms	268.10	196.09 ms	0.5815 ms	337.21
2.0	153.09 ms	0.5683 ms	269.38	199.0 ms	0.5810 ms	342.51
2.5	149.09 ms	0.5745 ms	259.51	190.73 ms	0.5865 ms	325.20
